# A mixed-methods protocol to explore psychological distress and psychosocial needs along the continuum of CKD care— a single healthcare network

**DOI:** 10.1186/s12882-025-04563-9

**Published:** 2025-11-18

**Authors:** Mathilde Paré, Norah Zola, Laëtitia Coudert, Julien-Carl Phaneuf, Roxanne Lavallée, Aude Caplette-Gingras, Catherine Fortier, Mohsen Agharazii

**Affiliations:** 1 Centre hospitalier universitaire (CHU) de Québec, Université Laval, Québec, QC Canada; 2 Endocrinology and Nephrology Research Program, CHU de Québec-Université Laval Research Center, Québec, QC Canada; 3 Faculty of Social Sciences, School of Psychology, Université Laval, Québec, QC Canada; 4 Department of Innovation in Medical Teaching, Faculty of Medicine, Université Laval, Québec, QC Canada; 5 Oncology Research Program, CHU de Québec-Université Laval Research Center, Québec, QC Canada; 6 Department of Kinesiology, Faculty of Medicine, Université Laval, Québec, QC Canada; 7 Department of Medicine, Faculty of Medicine, Université Laval, Québec, QC Canada

**Keywords:** Chronic kidney disease, Dialysis, Kidney transplant, Psychological distress, Anxiety, Depression, Psychosocial support

## Abstract

**Background:**

Chronic kidney disease (CKD) affects 8–16% of adults worldwide, including nearly 4 million Canadians. As the disease progresses, patients often experience a significant decline in quality of life (QoL), driven by disease-related symptoms and the burden of renal replacement therapies (RRT) such as dialysis and transplantation. In addition to physical symptoms, up to 55% of patients report psychological distress, including depression and anxiety. Despite this, mental health needs are frequently under-identified and insufficiently addressed in nephrology care. There is limited understanding of how patients experience and cope with this distress, how staff perceive their role in responding to it, and how healthcare environments support or hinder psychosocial care. This study seeks to address these gaps within a single integrated nephrology care network.

**Methods:**

This explanatory sequential mixed-methods study will be conducted in two phases. In the first phase, quantitative data will be collected from 200 patients with CKD and 40–50 nephrology staff members using validated questionnaires assessing psychological distress, anxiety, depression, and QoL. Group comparisons will assess differences across RRT modalities, and association analyses will explore relationships between psychological outcomes and demographic or clinical factors. In the second phase, semi-structured interviews and focus groups with patients and staff will explore experiences of psychological distress and care delivery in greater depth. Thematic analysis will be used for qualitative data. Integration of findings through triangulation will provide a comprehensive understanding of psychosocial needs and inform intervention development.

**Discussion:**

This study uses a mixed-methods design to investigate psychosocial needs in CKD care from both patient and provider perspectives. It aims to identify modifiable clinical and organizational factors—such as staff preparedness, referral practices, and systemic barriers—that shape the delivery of mental health support. Findings will inform the development of person-centered interventions and may guide broader models for integrating psychological care into chronic disease management.

**Status of trial:**

The study began in June 2024. Participant recruitment and data collection are ongoing and will continue until June 2026.

**Clinical Trial Number:**

Not applicable.

**Supplementary Information:**

The online version contains supplementary material available at 10.1186/s12882-025-04563-9.

## Background

Globally, chronic kidney disease (CKD) affects 8–16% of the adult population, posing a major public health challenge [1, 2]. In Canada, approximately 4 million individuals live with CKD [3, 4]. As CKD progresses, the burden of disease intensifies. Patients with advanced CKD or end-stage kidney disease (ESKD) often experience a significant decline in quality of life, comparable to that observed in other severe chronic illnesses such as cancer and heart failure [5–10]. This decline is driven by both the symptoms of the disease and the demands of renal replacement therapies, in the form of either in-center or home-based hemodialysis, peritoneal dialysis, or kidney transplantation. While these treatments are life-sustaining, they are frequently associated with pain, fatigue, sexual dysfunction, and other debilitating symptoms that impact physical, cognitive, psychological, and social well-being [9, 11].

Beyond its physical toll, CKD imposes a significant psychological burden, with studies documenting high levels of distress associated with both disease and treatment [12]. Depressive symptoms have been reported in up to 55% of individuals with CKD, and anxiety symptoms in up to 50%, across all treatment modalities [11, 13, 14]. Sources of psychological and existential distress identified, thus far, include lack of control over the disease and constant awareness of mortality. In parallel, studies have shown that patients use various coping strategies focusing on problem resolution and/or emotional regulation that can be adaptive or maladaptive depending on their unique circumstances [15–19].

Despite the growing body of evidence documenting these concerns, psychological needs remain insufficiently addressed in routine nephrology care. The availability and accessibility of psychosocial support services vary widely, and integration into standard clinical pathways is often limited. Furthermore, there is limited insight into how healthcare providers perceive their role in identifying and responding to patients’ mental health needs, or how they view existing support within their health care networks [20]. Understanding these perspectives is essential to inform the development of feasible and context-sensitive interventions that are acceptable to both patients and healthcare professionals. Such interventions have the potential not only to improve patient well-being and treatment adherence by reducing psychological burden, but also to enhance staff satisfaction, reduce burnout, and support workforce retention [21–24]. In turn, this may contribute to more sustainable models of kidney care and strengthen the overall quality and equity of service delivery.

Therefore, this mixed-method study seeks to explore the experience of psychological distress among patients with advanced CKD, as well as staff perceptions of mental health care delivery within a single integrated nephrology care network which provides nephrological care to patients who are scattered over a wide geographical territory. Furthermore, it aims to identify the characteristics of the health-care environment that influence the delivery of effective care, by integrating both patient and staff perspectives. By providing an in-depth understanding of psychological distress in CKD patients of our network, this study will lay the groundwork for the development of clinical and organizational interventions aiming to improve the well-being of patients across the different stages of disease and treatment modalities.

## Methods/design

### Design and method

**Fig. 1:**
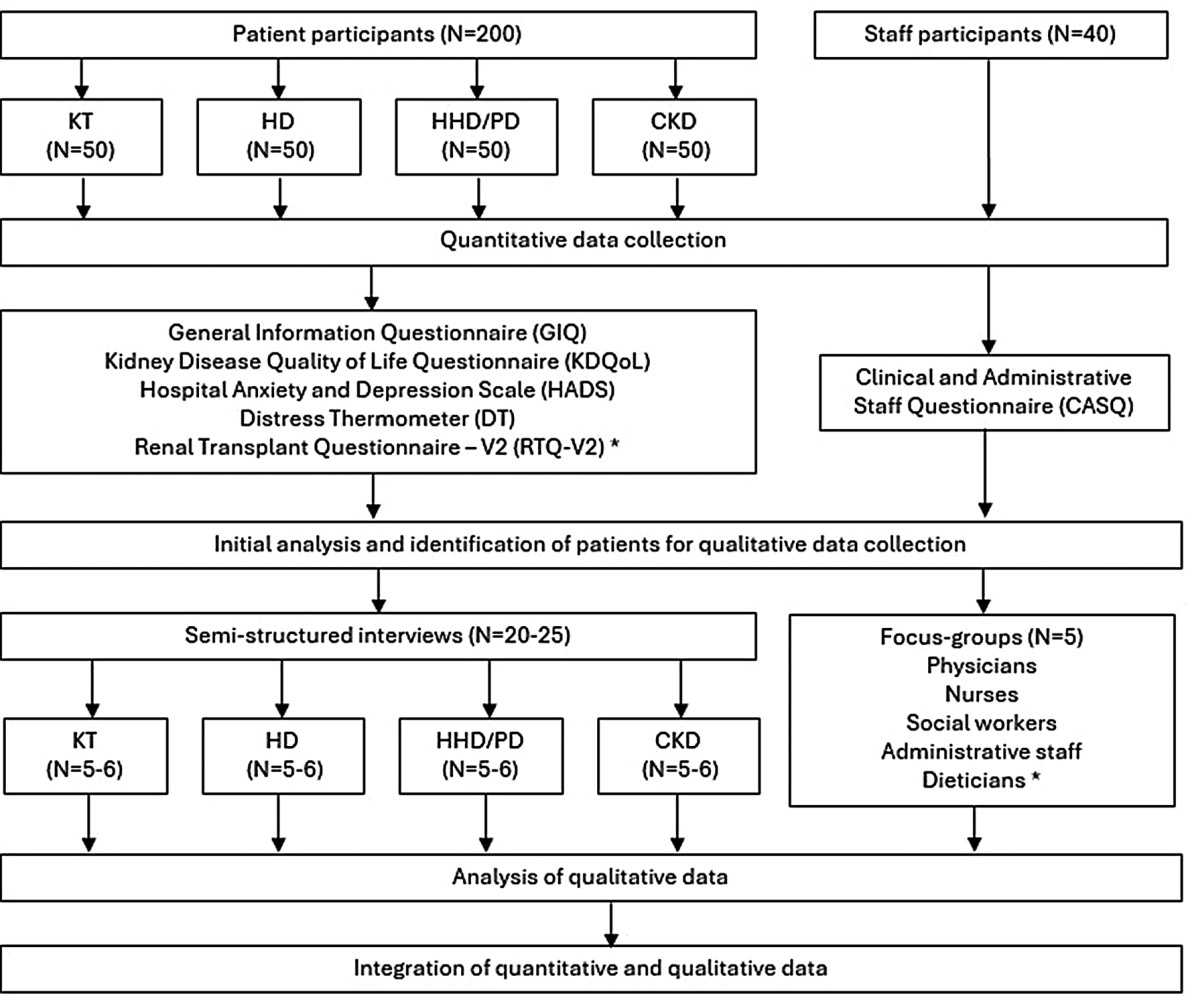
Flow diagram illustrating study protocol. CKD: chronic kidney disease pre-dialysis, HD : hemodialysis, HHD: home hemodialysis, KT: kidney transplant, PD: peritoneal dialysis. Dieticians ^*^ indicates that these participants will be distributed amongst other focus-groups, due to and insufficient number of dieticians amongst the studies health care network. RTQ-V2^*^ signifies that this questionnaire does not apply to all participants, but only to KT participants

This study employs a two-stage explanatory sequential cross-sectional mixed methods design, beginning with a quantitative study of both patient and staff perspectives through questionnaires and medical records, followed by a more detailed exploration of similar themes through qualitative analysis of individual interview and focus-group data [25]. A flow diagram of the study protocol design is provied in Fig. 1.

This design was selected to build a detailed understanding of experiences related to CKD in patients from a single care network, but with varied backgrounds, treatment modalities, and geographic dispersion. Quantitative data collection will be followed by an initial analysis describing the sample (e.g., gender, age, ethnic group) and assessing levels of psychological distress, anxiety, and depression. These results will inform purposive sampling for the qualitative phase, ensuring the inclusion of participants from diverse backgrounds and with varying degrees of distress, to obtain a balanced view of both risk and protective factors. The qualitative phase will then explore underlying mechanisms and contextual factors through in-depth interviews. This sequential design is deemed particularly appropriate given the study’s objective to generate a comprehensive understanding of care needs and system-level influences, while remaining feasible within a cross-sectional framework. It also supports the development of person-centered interventions by integrating measurable trends with nuanced experiential data.

This work constitutes the foundational phase of a broader research program guided by the Picker Principles of Person-Centered Care [26] and the United Kingdom Medical Research Council (UKMRC) Framework [27] for Developing and Evaluating Complex Interventions, both of which emphasize meaningful stakeholder engagement. Both patients, clinical staff, and administrators were approached to engage in this project, not only as participants, but as partners. Their input has informed the selection and adaptation of study questionnaires and the development of interview and focus-group guides to ensure relevance and acceptability. Sampling strategies were also refined with their feedback to capture diverse perspectives across treatment modalities. Upon completing data collection, they will be invited to contribute to interpretation and dissemination of findings, and to define priorities for and co-design subsequent interventions.

### Research questions

#### Main research question

What are the psychological support needs of patients with advanced CKD, and how can these needs be effectively addressed within an integrated care model?

#### Specific research questions centered on patients as participants

*Quantitative research questions*.

1. Does the level of psychological distress, anxiety, depression, and quality of life differ significantly across renal replacement therapy modalities?
2. What clinical, demographic, and social characteristics are associated with psychological distress and quality of life in CKD patients.

*Qualitative research questions*.

1. How do patients with CKD describe their day-to-day experience of living with the disease, particularly in relation to their psychological well-being?
2. How did patients with CKD experience the psychological or emotional support they received—or the absence of such support—along their care trajectory?
3. What do patients perceive as essential for promoting their well-being within their healthcare environment?

#### Specific research questions centered on staff as participants

Quantitative research questions

1. To what extent do staff perceptions, levels of satisfaction, and perceived preparedness regarding the provision of psychosocial support to CKD patients differ according to professional role, mental health training, years of experience, and frequency of patient contact?
2. What clinical and structural resources do staff find most useful for identifying and addressing psychological distress in patients with CKD?

Quanlitative research questions

1. How do healthcare and administrative staff perceive the psychological needs of patients with CKD in their care?
2. What experience do staff have with providing or facilitating support for patients’ psychological well-being?

### Setting

The study will be conducted within the CHU de Québec-Université Laval, the City of Québec’s network of teaching hospitals. This hospital network is the reference center for nephrology for the Eastern part of the province of Quebec, Canada, serving 2 million people, with a prevalent patient population of 500 pre-dialysis CKD patients, 1200 patients having received a kidney transplant and 300 hemodialysis and 90 home dialysis patients. While most patients treated at the institution are Caucasian French-Canadians, a significant minority of patients are from indigenous communities, and a smaller proportion from other ethnic or linguistic minorities.

The nephrology service multidisciplinary team is composed of nephrology unit nursing staff, specialized clinic nursing staff, pharmacists, dieticians, social workers, staff nephrologists, nephrology fellows (i.e. nephrologists pursuing additional training), nephrology trainees, administrative personnel, and hospital coordinators.

### Study population and sampling

#### Patients

Patients with CKD will be recruited based on their current treatment modality, categorized as follows: 1) pre-dialysis CKD, 2) in-center hemodialysis, 3) home dialysis (including both peritoneal and home hemodialysis), and 4) kidney transplantation. A total of 200 patients —50 from each treatment group—will be recruited for the quantitative component of the study using standardized questionnaires.

As sampling progresses, patient demographic information will be reviewed and purposive sampling of patients from underrepresented groups will be undertaken. From this sample, 20 to 25 participants will be purposively selected to take part in individual semi-structured interviews exploring their lived experiences.

Eligible patient participants are adults (≥ 18 years) with CKD, defined as an estimated glomerular filtration rate (eGFR) < 60 mL/min/1.73 m² or as having received a kidney transplant. The choice of eGFR cut-off was made, as stage 3 CKD with eGFR < 60 patients mL/min/1.73 m² has been associated with increased cardiovascular mortality and hospitalization [28], as well as more frequent follow-ups, blood works and an increasing number of medications. Patients will be excluded if they are unable to provide informed consent or to complete study questionnaires due to cognitive, emotional, or language limitations, even with the assistance of research staff, an interpreter, or an Indigenous liaison officer.

Recruitment will follow a quota-based convenience sampling strategy, with equal numbers of participants recruited from each treatment group to ensure balanced comparisons. Eligible patients will be approached consecutively during routine nephrology clinic visits. Initial contact will be made by a clinic staff member, after which interested patients will be referred to the research team for informed consent. Participants may complete the questionnaires on-site or take them home to allow time for reflection before contacting the research team to enroll or with additional questions.

#### Staff

All clinical and administrative nephrology staff are eligible to participate, regardless of job title. Staff recruitment will follow a proportionate stratified sampling approach, ensuring representation of professional roles in accordance with their relative size within the nephrology workforce [29]. The project will be introduced to staff of different job titles by their coordinator or a research team member (RL, NZ), either directly in clinical settings or through email. Consent forms and questionnaires will be distributed in that same way, with information on how to return them should they wish to participate.

### Samples size calculation

#### Quantitative sample

**Patient participants**: As suggested in the mixed-methods literature, a sample size of 30 participants per group is generally sufficient to allow for regression analyses based on questionnaire data [30, 31]. Sample size analysis shows that a total sample size of 200 patients (4 groups of 50 cases) ensures an 84% power for the F-test of ANOVA to detect a moderate effect size of f = 0.25 with an alpha error of 0.05.

**Nephrology staff participants**: Given the *survey* nature of the staff questionnaire and the fact that it has not been externally validated, a minimal sample size of *N* = 40 staff members has been agreed upon based on similar designs within the literature. Given the accessibility of staff members and their numbers, it is likely that by approaching 60 staff members, between 40 and 50 participants will be recruited.

#### Qualitative sample

**Patient participants**: Sample size will be guided by the principle of data saturation, the point at which no new thematic insights emerge with additional data collection [32]. Given the expected diversity of experiences across disease stages and treatment modalities, we plan to include approximately 20–25 patients living with CKD in individual interviews. Individual interviews were chosen to allow participants to share personal information relative to their mental health without being concerned about other participants’ judgment or confidentiality.

**Nephrology staff participants**: Profession-specific focus groups will be conducted amongst nephrology staff to enhance homogeneity and allow for deeper, role-specific discussion grounded in shared clinical realities. Focus groups were chosen to facilitate the co-construction of ideas and maximize participation in clinical contexts where time is scarce. We anticipate conducting 1 focus group per professional subgroup (e.g., nurses, physicians), each including 5–8 participants [32]. Individual interviews will also be offered for health care professionals who are unable to participate in a focus-group session or for those from professional subgroups with limited numbers of participants (e.g., fewer than 3 individuals such as nutritionists and social workers). When appropriate, these participants could also be integrated into interprofessional focus groups. This decision will be based on logistical considerations and participant preferences, while ensuring the integrity of the data collection process.

### Instruments

#### Quantitative data collection

During the initial study visit, all participating patients will complete a battery of questionnaires, either self-administered or with assistance from research staff, depending on individual needs.

- **General Information Questionnaire (GIQ)**: This questionnaire was adapted from a tool developed by Taylor et al. as part of a mixed-methods protocol exploring psychological and emotional support needs in end-stage renal disease and published in *BMJ Open* under a CC BY 4.0 license [20]. It assesses distress, psychosocial concerns, and perceived support within the healthcare setting. The tool has been translated and the language adapted for French (Canadian) speaking participants. Additional questions were included to identify relevant demographic and clinical variables that may affect psychological distress, well-being and quality of life. The translated and adapted version is presented in Supplemental file A.
- **Kidney Disease Quality of Life Instrument (KDQOL)**: The KDQOL is a well-validated disease-specific measure developed to assess health-related quality of life in individuals with CKD and those receiving dialysis [33]. The French translation has also been validated [34]. It incorporates both general and kidney-specific domains. This questionnaire will be administered to all patient participants, including those with a kidney transplant, to allow for cross-group comparisons.
- **Hospital Anxiety and Depression Scale (HADS)**: The HADS is a 14-item instrument that measures symptoms of anxiety and depression and is particularly suitable for use in medically ill populations, as it minimizes confounding by physical symptoms [35]. The questionnaire has been validated for use in ESKD patients [36, 37], and the French translation has also been validated [38].
- **Distress Thermometer (DT)**: The DT is a brief screening tool that assesses overall psychological distress and includes a checklist of common contributing issues (physical, emotional, social). This National Comprehensive Cancer Network Tool has been widely validated across chronic disease populations and been used in French speaking cohorts of patients, as well as CKD cohorts of patients [39, 40].
- **Renal Transplant Questionnaire, Version 2 (RTQ-V2)**: The RTQ-V2 is a transplant-specific quality of life instrument validated in kidney transplant populations [41]. It assesses unique concerns related to immunosuppression, graft function, and transplant-related lifestyle factors. This instrument will be administered only to patients who have received a kidney transplant, in addition to the KDQOL.
- **Clinical and Administrative Staff Questionnaire (CASQ)**, adapted and translated from Taylor et al. [20]. This questionnaire assesses staff perceptions of psychological distress among patients with CKD, as well as unmet psychosocial needs and barriers for patients to access appropriate mental health support, in line with the study’s research questions. Its methodological properties are presented in the original article. The adapted questionnaire is provided in supplemental file B.

Additionally, relevant demographic and clinical data will be extracted from patients’ electronic medical records to help identify potential determinants of psychological distress and well-being among individuals with CKD. Variables to be examined will include the nature and number of comorbid medical conditions, as well as key biological parameters such as hemoglobin levels, acid-base balance, and markers of uremia. Psychiatric history and current use of psychoactive medications will also be considered covariates. Finally, dialysis-related variables — including dialysis vintage, history of previous renal replacement therapy modalities, and prior kidney transplant loss — will be included in the analysis.

#### Qualitative data collection

Qualitative data will be collected through individual semi-structured interviews and profession-based focus groups, conducted by research team members trained in psychosocial interviewing and in the study’s objectives. Ideally, the same interviewer will conduct all sessions to ensure consistency; if this is not possible, another interviewer with equivalent training will serve as substitute. Interviewers will not be known to patients or staff outside the research context and are not part of the clinical team, to minimize the risk of censoring by participants.

Sessions will be held in person whenever possible, in a quiet private room at the research center across from the hospital to facilitate participation. For data security and sound quality, two recording devices will be used. If in-person meetings are not feasible, secure institutional Microsoft Teams^®^ accounts will be used for videoconferencing and recording. All audio files and anonymized transcripts will be stored on a secure research center server, with a second team member validating transcription accuracy. In addition, transcripts will be reviewed after each interview by JCP, the team’s most experienced qualitative data collector, to ensure rigor and consistency.

Individual interviews are expected to last 50–75 min, without a fixed time limit; participants may end the discussion at any time, and redundancy in topics will prompt closure. Focus groups with staff are expected to last 90–120 min, also without a fixed time limit; they will be moderated by a trained facilitator while another team member collects field notes on non-verbal communication. Interview and focus-group guides are provided in Supplemental Files C and D, respectively. In line with the principles of semi-structured interviewing, the questions were designed to remain open and non-directive, allowing participants to share their concrete experiences without undue influence from interviewers. The guides will be iteratively reviewed and refined as interviews progress, and interviewers will be trained to use probing questions that encourage depth and clarity without interpreting or steering participants’ testimony.

### Data analysis

#### Quantitative data analysis

Statistical analyses will provide a description of distress and well-being scores measured using quantitative questionnaires (GIQ, KDQOL, HADS, DT, RTQ-V2) from patients with kidney disease. An initial descriptive analysis will summarize demographic and clinical characteristics, along with distress and well-being scores. These results will guide purposive sampling for the qualitative phase by ensuring diversity in backgrounds and variation in distress levels, so that both risk and protective factors are represented. Further analysis will determine whether differences exist between subgroups of kidney disease patients and examine the effect of various underlying sociodemographic and clinical factors. Statistical analyses will be conducted using SAS Statistical Software v.9.4 (SAS Institute, Cary, NC, USA),

Distress and well-being scores, as well as other continuous variables, will be presented as means, standard deviations, 95% confidence intervals, medians, and interquartile ranges. ANOVA (or Kruskal-Wallis) with subsequent t-test (or Mann-Whitney U test) will be used to assess differences across categories of renal replacement therapy. Categorical variables will be described using frequencies and percentages. Chi-square or Fisher’s exact tests will be used to assess differences across categories of renal replacement therapy. Associations between distress/well-being scores, and socio-demographical, and clinical parameters will be explored using univariate and multivariate regression models. Tukey-Kramer adjustment will be applied for multiple comparisons. A two-tailed p-value of < 0.05 is considered statistically significant.

#### Qualitative data analysis

Data from the semi-structured interviews and focus groups will be analyzed using thematic analysis, following the approach outlined by Braun and Clarke [42]. Recordings will be transcribed and coded both manually and using NVivo (QSR International Pty Ltd., Melbourne, Australia). First, thematic analysis of interview transcripts will be conducted manually on an ongoing basis as interviews are completed, and repeated once the full dataset has been collected. This iterative process will allow preliminary coding to inform subsequent data collection and facilitate early identification of emerging themes. Data saturation will be monitored throughout the analysis, defined as the point at which no new codes or themes arise from successive transcripts. To enhance rigor, the coding process will be revisited once all interviews are completed to ensure consistency and comprehensiveness in theme development.

### Data interpretation and integration

This is a cross-language study which will be conducted in French, using questionnaires that have either been validated for use in French speakers, or in-house questionnaires developed by Quebec French-speaking research staff. Data will be disseminated locally in French, but to broader audiences in English (articles, conferences). Recognizing that translation is an interpretive act that can affect meaning, we will ensure that data are initially analyzed in the original language (coding and thematic analysis) to preserve linguistic and cultural nuance. Key quotations will be translated by bilingual team members, with translator annotations to contextualize culturally specific terms or concepts. When possible, collaborative review will be employed to enhance interpretive fidelity. This approach aims to maintain the authenticity of participants’ voices while ensuring clarity and coherence in reporting for an international audience [43–45].

Quantitative and qualitative findings will be integrated during the interpretation phase to provide a comprehensive understanding of psychological distress in patients with chronic kidney disease and the perspectives of nephrology staff. Both data strands will be given equal weight in the analysis. Triangulation will be used to identify points of convergence, divergence, and complementarity between questionnaire results and interview or focus group themes. This approach ensures that statistical patterns are enriched by contextual and experiential insights, thereby informing the development of person-centered interventions grounded in both empirical trends and lived realities [25].

Quantitative and qualitative findings will be integrated during the interpretation phase using an Explanatory Sequential Design Joint Display [46], as exemplified in Finley et al.’s work on relationship quality and patient care [47]. This display will visually align quantitative results (e.g., levels of distress, anxiety, and quality of life by treatment modality) with qualitative themes from interviews and focus groups. By presenting these findings side by side, the display will make explicit where the two strands converge, diverge, or offer complementary insights, thereby supporting the generation of transparent and integrated interpretations.

In addition, we will employ a “following a thread” approach [48]. This strategy involves taking a key finding from one dataset and systematically tracing it across the others. For example, if quantitative analyses reveal higher distress among transplant patients, we will examine how this theme emerges in patient interviews and staff focus groups. This iterative process helps build a multi-layered understanding of psychological distress and the contextual factors that shape it.

Where it adds value, we may also use limited quantitization [49]. This may include reporting the frequency with which certain themes appear in the qualitative data (e.g., fatigue, isolation, or coping strategies) to facilitate structured comparison with quantitative patterns. Such transformation will be applied selectively and reported transparently, with the perspectives of both patients and staff kept central to the interpretation.

Finally, integration will be strengthened by applying the triangulation protocol described by Farmer et al. (2006) [50]. This six-step framework involves sorting findings into comparable segments, applying a convergence coding scheme (agreement, partial agreement, silence, dissonance), assessing overall convergence and completeness, comparing interpretations across the research team, and feeding results back to stakeholders for clarification. By following this structured and auditable process, we will ensure that both convergences and divergences across datasets are identified, explored, and meaningfully interpreted, thereby enhancing the rigor and trustworthiness of the study’s conclusions.

## Discussion

This study will employ a sequential explanatory mixed methods design to explore psychological distress among patients with advanced CKD and to understand how mental health care is perceived and addressed by nephrology staff. By integrating quantitative data on distress prevalence, anxiety, depression, and quality of life with qualitative insights into the lived experience of patients and the perspectives of staff, this study will provide a comprehensive understanding of psychological needs within a single integrated nephrology care network.

The study is innovative in its dual focus: it will assess the extent of psychological distress experienced by patients while also exploring systemic and professional-level factors (training, years of experience, etc.) that shape the capacity to address this distress in practice. High levels of psychological distress, including anxiety and depression, are well documented in individuals with CKD, with prevalence rates reaching 55% depending on treatment modality [11, 13, 14]. However, psychological needs often remain under-identified and insufficiently addressed in routine nephrology care. This project addresses that gap by incorporating both patient and provider perspectives, which is essential for the design of interventions that are both feasible and contextually appropriate [20].

This is one of the first studies in Quebec – a majority French speaking population in Canada – to apply a mixed-methods approach to simultaneously capture both patient and provider experiences with psychosocial aspects of CKD care. It is also among the few that aim to identify modifiable characteristics of the healthcare environment—such as staff preparedness, time constraints, and referral pathways—that influence the integration of mental health care into nephrology services.

Findings from this research will contribute to the development of targeted, context-sensitive interventions aimed at improving the identification and management of psychological distress in patients with CKD. These interventions may include validated screening tools enhanced access to psychosocial professionals and strengthened interdisciplinary collaboration. In turn, such improvements could lead to better treatment adherence, patient and staff satisfaction, as well as reduced reliance on acute care services [21, 22, 51].

In addition to its clinical impact, this study will advance theoretical understanding in health psychology by contributing to a contextual model of psychological distress in CKD and reinforcing person-centered care models [26]. Insights gained may be transferable to other chronic disease populations and inform broader strategies for system-wide transformation in chronic care delivery [27].

The use of both quantitative and qualitative methods will enable a more nuanced and holistic interpretation of patient and staff experiences. Triangulation of findings will ensure that numerical trends are complemented and enriched by in-depth narratives, enhancing the validity and utility of the results. This balanced integration of data types is critical to ensure that intervention development is grounded not only in prevalence estimates but also in the lived realities of those affected.

However, some limitations must be acknowledged. The use of quota-based convenience sampling in the patient population may introduce selection bias, as individuals who access care more frequently or are more engaged in their care may be overrepresented [52]. Additionally, while focus groups offer rich data on shared experiences among staff, they may limit disclosure of sensitive or dissenting views [32]. Finally, the translation of qualitative data from French to English for publication purposes presents the risk of subtle meaning loss; to mitigate this, bilingual researchers will be involved in translation and review [44, 45].

Despite these limitations, this study will generate practical knowledge that can inform the co-design of sustainable, person-centered interventions to better support the mental health and well-being of individuals living with CKD.

## Supplementary Information

Below is the link to the electronic supplementary material.

Supplementary Material 1

## Data Availability

No datasets were generated or analysed during the current study.

## Abbreviations

CASQ: Clinical and Administrative Staff Questionnaire
CHU: Centre Hospitalier Universitaire
CHUQ-UL: CHU de Québec–Université Laval
CKD: Chronic Kidney Disease
DT: Distress Thermometer
eGFR: Estimated Glomerular Filtration Rate
ESKD: End-Stage Kidney Disease
GIQ: General Information Questionnaire
HADS: Hospital Anxiety and Depression Scale
KDQOL: Kidney Disease Quality of Life Instrument
RRT: Renal Replacement Therapy
RTQ-V2: Renal Transplant Questionnaire, Version 2
UKMRC: United Kingdom Medical Research Council
