# Supplementary material for: A mixed-methods protocol to explore psychological distress and psychosocial needs along the continuum of CKD care— a single healthcare network
Source: BMC Nephrol. 2025 Nov 18;26:644. doi: 10.1186/s12882-025-04563-9 (PMC12625347; doi:10.1186/s12882-025-04563-9)
Supplement: Supplementary file 1 — Supplementary Material 1 [file 12882_2025_4563_MOESM1_ESM.docx]

**SUPPLEMENTAL FILE A:** General information questionnaire

**Questionnaire d’informations générales pour les personnes atteintes d’une maladie rénale (QIG)**

Répondez aux questions suivantes au meilleur de vos connaissances.

Si vous ne comprenez pas une question, n’hésitez pas à demander de l’aide à un proche ou un membre de l’équipe de recherche.

Si vous n’êtes pas à l’aise de répondre à une question, sentez-vous libre de ne pas y répondre.

Sachez toutefois que l’ensemble des réponses resteront confidentielles.

1. Nom : _____________________
2. Prénom : ______________________
3. Âge : ____________________
4. Date de naissance : _________________________
5. Sexe à la naissance :

- Homme
- Femme
- Ne sait pas
- Ne veut pas répondre

1. Identité de genre :

- Homme
- Femme
- Ne veut pas répondre
- Autre, précisez si vous le souhaitez : ___________________________

1. État civil :

- Marié(e)/Union libre
- Séparé(e)/Divorcé(e)
- Célibataire
- Veuf ou veuve

1. Habitez-vous avec d’autres personnes ?

- Oui
- Non

Si vous avez répondu « oui » à la question précédente, qui sont **les personnes avec qui vous habitez** ?

- Membre(s) de la famille
- Conjoint(e)
- Ami(es)
- Aidant(e) naturel(le)
- Autre : ___________________________

1. Quel est le **dernier niveau de scolarité que vous avez complété** ?

- Primaire
- Secondaire
- Secondaire professionnel
- Collégial pré-universitaire
- Collégial technique
- Universitaire, 1er cycle
- Universitaire, 2er cycle
- Universitaire, 3er cycle
- Autre : ________________

1. Quelle est votre **occupation principale** **actuelle** ?

- Études
- Travail à temps partiel
- Travail à temps complet
- Travail familial non-rémunéré
- Retraite
- Congé de maladie/arrêt de travail
- Invalidité permanente
- Sans travail/recherche d’emploi
- Autre : ________________

1. À combien estimez-vous votre **revenu familial annuel brut** (avant déduction des impôts) ?

- 20 000$ et moins         q 60 001$ à 80 000$     q 120 001 à 140 000$
- 20 001$ à 40 000$       q 80 001$ à 100 000$         q 140 001$ et plus
- 40 001$ à 60 000$       q 100 001$ à 120 000$       q Je ne sais pas/je refuse de répondre

1. Quelle langue parlez-vous avec le plus de facilité ?

- Français
- Anglais
- Espagnol
- Innu-aimun
- Autre : __________________

1. À quel groupe ethnique appartenez-vous ?  SVP, cochez la réponse la plus proche de votre situation et/ou précisez.

- Caucasien (blanc)
  - Né au Canada
  - Né en dehors du Canada
    - Précisez : _________________
- Communauté autochtone du Canada
  - Précisez : _________________
- Communauté autochtone hors du Canda
  - Précisez : _________________
- Noir
  - D’origine africaine
  - D’origine des caraïbes
  - Autre, précisez : __________________
- Asiatique
  - Asie centrale
  - Asie de l’Est
  - Asie du Sud-Est
  - Asie du Nord
  - Asie de l’Ouest ou moyen orient
  - Autre, précisez : __________________
- Arabe
  - Précisez : __________________
- Perse
- Autre groupe ethnique :
- Précisez : __________________

1. Avez-vous déjà consulté un professionnel pour des problématiques psychologiques ?

- Non
- Oui

Si oui, précisez :

- Psychologue
- Psychiatre
- Médecin de famille
- Psychoéducateur
- Travailleur social
- Autre, précisez, svp : _______________

1. Est-il important que le personnel soignant de néphrologie **apporte un soutien aux patients quand ils ressentent des émotions et des sentiments négatifs** persistants à cause de leur maladie rénale ?

(Veuillez remplir ou faire un X sur le cercle **O** qui décrit le mieux l’importance que y accordez.)

| **Aucunement important** | |  |  |  |  |  |  |  | **Vraiment important** | |
| --- | --- | --- | --- | --- | --- | --- | --- | --- | --- | --- |
| **0** | **1** | **2** | **3** | **4** | **5** | **6** | **7** | **8** | **9** | **10** |
| O | O | O | O | O | O | O | O | O | O | O |

1. D’après votre propre expérience, **êtes-vous satisfait par l’offre de service en psychologie** proposée par le service de néphrologie ?

(Veuillez remplir ou faire un X sur le cercle **O** qui décrit le mieux l’importance que y accordez.)

| **Aucunement satisfait** | |  |  |  |  |  |  |  | **Vraiment satisfait** | |
| --- | --- | --- | --- | --- | --- | --- | --- | --- | --- | --- |
| **0** | **1** | **2** | **3** | **4** | **5** | **6** | **7** | **8** | **9** | **10** |
| O | O | O | O | O | O | O | O | O | O | O |

Si vous le souhaitez, veuillez **préciser en quoi vous êtes satisfait ou insatisfait de l’offre de service :**

1. Vous trouverez ci-dessous **des commentaires faits par d’autres patients sur le soutien** qu’ils reçoivent de la part du personnel soignant de néphrologie.

Veuillez indiquer **dans quelle mesure vous êtes d’accord ou non** avec les affirmations suivantes :

|  | **Aucunement d’accord** | | | | |  | |  | |  | | **Vraiment d’accord** | | | |
| --- | --- | --- | --- | --- | --- | --- | --- | --- | --- | --- | --- | --- | --- | --- | --- |
| Je peux discuter de mes inquiétudes ou de mes craintes avec le personnel soignant. | 0 | 1 | 2 | 3 | 4 | | 5 | | 6 | | 7 | | 8 | 9 | 10 |
| J’ai parfois l’impression que le personnel soignant traite plus ma maladie que moi, la personne que je suis dans ma globalité. | 0 | 1 | 2 | 3 | 4 | | 5 | | 6 | | 7 | | 8 | 9 | 10 |
| Le personnel soignant n’a pas le temps de m’écouter. | 0 | 1 | 2 | 3 | 4 | | 5 | | 6 | | 7 | | 8 | 9 | 10 |
| J’ai l’impression que le personnel soignant se soucie vraiment de moi. | 0 | 1 | 2 | 3 | 4 | | 5 | | 6 | | 7 | | 8 | 9 | 10 |
| Je n’ai pas besoin du soutien du personnel soignant, car je suis bien soutenu(e) par ma famille et/ou mes amis. | 0 | 1 | 2 | 3 | 4 | | 5 | | 6 | | 7 | | 8 | 9 | 10 |
| La façon dont le personnel soignant me traite est très réconfortante. | 0 | 1 | 2 | 3 | 4 | | 5 | | 6 | | 7 | | 8 | 9 | 10 |
| J’aimerais parler de mes inquiétudes ou de mes craintes à un membre du personnel soignant, mais je ne sais pas à qui m’adresser. | 0 | 1 | 2 | 3 | 4 | | 5 | | 6 | | 7 | | 8 |  |  |

1. Réfléchissez à **la façon dont** **vous faites face à votre maladie et à votre traitement.**

Veuillez indiquer **dans quelle mesure vous êtes d’accord ou non** avec les affirmations suivantes :

|  | **Aucunement d’accord** | | | | |  | |  | | **Vraiment d’accord** | | | | |  |
| --- | --- | --- | --- | --- | --- | --- | --- | --- | --- | --- | --- | --- | --- | --- | --- |
| Je n’ai aucun problème ou aucune difficulté à faire face à ma maladie. | 0 | 1 | 2 | 3 | 4 | | 5 | | 6 | | 7 | 8 | 9 | 10 | |
| Je crains que mon état ne s’aggrave. | 0 | 1 | 2 | 3 | 4 | | 5 | | 6 | | 7 | 8 | 9 | 10 | |
| Je parviens à maîtriser mes sentiments négatifs à l’égard de ma maladie et de mon traitement. | 0 | 1 | 2 | 3 | 4 | | 5 | | 6 | | 7 | 8 | 9 | 10 | |
| J’ai du mal à accepter ma maladie. | 0 | 1 | 2 | 3 | 4 | | 5 | | 6 | | 7 | 8 | 9 | 10 | |
| J’essaie de penser positivement à ma maladie et à mon traitement. | 0 | 1 | 2 | 3 | 4 | | 5 | | 6 | | 7 | 8 | 9 | 10 | |
| Je me sens nerveux à l’idée de ce qui va se passer dans le futur. | 0 | 1 | 2 | 3 | 4 | | 5 | | 6 | | 7 | 8 | 9 | 10 | |
| Je suis satisfait(e) de la façon dont je fais face à ma maladie et à mon traitement. | 0 | 1 | 2 | 3 | 4 | | 5 | | 6 | | 7 | 8 | 9 | 10 | |
| Je crains de devenir plus angoissé(e) à l’avenir. | 0 | 1 | 2 | 3 | 4 | | 5 | | 6 | | 7 | 8 | 9 | 10 | |
| Je me sens capable de faire face à toute difficulté liée à ma maladie et à mon traitement. | 0 | 1 | 2 | 3 | 4 | | 5 | | 6 | | 7 | 8 |  |  | |

- Vous trouverez ci-dessous une **liste des différents types de soutien** que d’autres services de néphrologie offrent aux patients. Pour chaque type de soutien, **veuillez indiquer dans quelle mesure il vous intéresse**.

|  | **Aucunement d’accord** | | | | |  |  | **Vraiment d’accord** | | | |
| --- | --- | --- | --- | --- | --- | --- | --- | --- | --- | --- | --- |
| Du temps pour discuter de mes émotions ou sentiments avec le personnel soignant. | 0 | 1 | 2 | 3 | 4 | 5 | 6 | 7 | 8 | 9 | 10 |
| Des formations en personne pour apprendre à gérer mes pensées et mes sentiments négatifs. | 0 | 1 | 2 | 3 | 4 | 5 | 6 | 7 | 8 | 9 | 10 |
| Parler de ma situation de vie avec un psychologue ou un intervenant spécialisé en santé mentale, lors de ma visite à l’hôpital. | 0 | 1 | 2 | 3 | 4 | 5 | 6 | 7 | 8 | 9 | 10 |
| Une discussion amicale en tête-à-tête avec un autre patient organisée par le service de néphrologie. | 0 | 1 | 2 | 3 | 4 | 5 | 6 | 7 | 8 | 9 | 10 |
| Une formation en ligne sur la gestion de mes sentiments et de mes humeurs. | 0 | 1 | 2 | 3 | 4 | 5 | 6 | 7 | 8 | 9 | 10 |
| Pouvoir indiquer à l’avance, sur une feuille remise par le personnel, les enjeux que je souhaite aborder lors de ma visite médicale. | 0 | 1 | 2 | 3 | 4 | 5 | 6 | 7 | 8 | 9 | 10 |
| Un programme personnel d’activité physique conçu pour les patients comme moi. | 0 | 1 | 2 | 3 | 4 | 5 | 6 | 7 | 8 | 9 | 10 |
| Un programme d’activités sur les saines habitudes de vie conçu pour des patients comme moi. | 0 | 1 | 2 | 3 | 4 | 5 | 6 | 7 | 8 | 9 | 10 |
| Un programme d’ateliers artistique ou de détente conçu pour des patients comme moi. | 0 | 1 | 2 | 3 | 4 | 5 | 6 | 7 | 8 | 9 | 10 |
| Un groupe de soutien en ligne avec d’autres patients souffrants de maladies rénales. | 0 | 1 | 2 | 3 | 4 | 5 | 6 | 7 | 8 | 9 | 10 |
| Un groupe de soutien en présentiel avec d’autres patients souffrants de maladies rénales. | 0 | 1 | 2 | 3 | 4 | 5 | 6 | 7 | 8 | 9 | 10 |

Le cas échéant, **veuillez indiquer tout autre type de soutien que vous souhaiteriez que le service de néphrologie vous apporte** afin de répondre à vos besoins psychologiques et psychosociaux.

**SUPPLEMENTAL FILE B:** Clinical and Administrative Staff Questionnaire CASQ

**Questionnaire à destination du personnel clinico-administratif des services de néphrologie et de l’administration du CHU de Québec - Université Laval (QPCA).**

 1. Quelle proportion de personnes atteintes de maladie rénale chronique dans les services de néphrologie pensez-vous être en détresse psychologique ? *(Veuillez cocher, svp)*

| **Moins de 10%** | 10-20% | 21-30% | 31-40% | 41-50% | 51-60% | 61-70% | 71-80% | Plus de 80% | **Je ne sais pas** |
| --- | --- | --- | --- | --- | --- | --- | --- | --- | --- |
| O | O | O | O | O | O | O | O | O | O |

2. Dans quelle mesure croyez-vous que la maladie rénale contribue à la détresse psychologique vécue par les personnes qui fréquentent les services de néphrologie ? *(Veuillez cocher, svp)*

| **Pas du tout** | |  |  |  |  |  |  |  | **J’en suis**  **convaincue** | |
| --- | --- | --- | --- | --- | --- | --- | --- | --- | --- | --- |
| 0 | 1 | 2 | 3 | 4 | 5 | 6 | 7 | 8 | 9 | 10 |
| O | O | O | O | O | O | O | O | O | O | O |

3. Quelles sont les difficultés émotionnelles principales qui vous ont été rapportées ou que vous avez observées chez les personnes suivis en néphrologie ? *(Cocher toutes celles qui s’appliquent et/ou décrivez vos observations)*

- Humeur dépressive
- Anxiété
- Angoisse
- Difficultés d’adaptation
- Colère
- Frustration
- Désespoir / perte de sens de la vie
- Culpabilité
- Autre, précisez : __________________

 4. Selon vous, quels sont les facteurs de stress spécifiques à la maladie rénale chronique et sa prise en charge qui contribuent à la détresse des personnes atteintes de maladie rénale chronique ? *(Cochez tous ceux qui s’appliquent et/ou détaillez vos observations.)*

- Le temps nécessaire pour effectuer les traitements
- La logistique des déplacements vers l’hôpital
- La perte de revenu et l’insécurité financière
- Porter un diagnostic de maladie chronique
- Les symptômes physiques en lien avec la maladie
- Les dysfonctions sexuelles
- L’impact de la maladie chronique sur leurs proches
- L’altération de l’image de soi
- Les interactions avec les professionnels de la santé
- Les traitements de dialyse
- Les incertitudes face au devenir de leur maladie
- Autre, précisez : __________________

5. Pensez-vous qu'il soit bénéfique pour la santé globale des personnes atteintes de maladie rénale chronique que les services de néphrologie les aide à adresser leur détresse psychologique ? *(Veuillez cocher, svp)*

| **Pas du tout bénéfique** | |  |  |  |  |  |  |  | **Très bénéfique** | |
| --- | --- | --- | --- | --- | --- | --- | --- | --- | --- | --- |
| 0 | 1 | 2 | 3 | 4 | 5 | 6 | 7 | 8 | 9 | 10 |
| O | O | O | O | O | O | O | O | O | O | O |

6. Dans quelle mesure êtes-vous satisfait du soutien que les services de néphrologie apporte aux personnes atteintes de maladie rénale chronique afin d’adresser leur détresse psychologique ? *(Veuillez cocher, svp)*

| **Pas du tout satisfaisant** | |  |  |  |  |  |  |  | **Très satisfaisant** | |
| --- | --- | --- | --- | --- | --- | --- | --- | --- | --- | --- |
| 0 | 1 | 2 | 3 | 4 | 5 | 6 | 7 | 8 | 9 | 10 |
| O | O | O | O | O | O | O | O | O | O | O |

7. Vous trouverez ci-dessous une liste de déclarations faites par d'autres membres du personnel clinico-administratif de néphrologie concernant leur **responsabilité** dans l'identification et la réponse aux besoins des personnes atteintes de maladie rénale chronique en lien avec leur détresse psychologique. Dans quelle mesure êtes-vous d'accord ou non avec chaque affirmation ? *(Veuillez cocher, svp)*

|  | **Fortement en désaccord** | | |  |  |  |  |  | **Fortement en accord** | | |
| --- | --- | --- | --- | --- | --- | --- | --- | --- | --- | --- | --- |
|  | 0 | 1 | 2 | 3 | 4 | 5 | 6 | 7 | 8 | 9 | 10 |
| C'est la responsabilité des services de néphrologie, mais pas ma responsabilité individuelle comme membre du personnel. | O | O | O | O | O | O | O | O | O | O | O |
| C'est la responsabilité d'autres professionnels,  tels que les médecins généralistes, les psychothérapeutes ou les bénévoles et employés d’organismes communautaires. | O | O | O | O | O | O | O | O | O | O | O |
| Cela fait partie de ma description de tâches. | O | O | O | O | O | O | O | O | O | O | O |
| Je n'ai pas été formé.e pour identifier la détresse psychologique des patients suivis en néphrologie. | O | O | O | O | O | O | O | O | O | O | O |
| Je trouve que c'est un rôle épanouissant (d’identifier et adresser la détresse des patients). | O | O | O | O | O | O | O | O | O | O | O |
| Il n’y a pas de bénéfice à assumer cette  responsabilité, car elle n'est  pas récompensée en reconnaissance/salaire/promotion. | O | O | O | O | O | O | O | O | O | O | O |
| Je reconnais les avantages potentiels pour mes patients si je devais/pouvais assumer ce rôle. | O | O | O | O | O | O | O | O | O | O | O |
| C’est une partie importante de mon travail. | O | O | O | O | O | O | O | O | O | O | O |

6. Vous trouverez ci-dessous une liste de déclarations faites par d'autres membres du personnel clinico-administratif de néphrologie sur leur **capacité** à identifier et à répondre aux besoins des personne atteintes de maladie rénale chronique en lien avec leur détresse psychologique. Dans quelle mesure êtes-vous d'accord ou non avec chaque affirmation ? *(Veuillez cocher, svp)*

|  | **Fortement en désaccord** | | |  |  |  |  |  | **Fortement en accord** | | |
| --- | --- | --- | --- | --- | --- | --- | --- | --- | --- | --- | --- |
|  | 0 | 1 | 2 | 3 | 4 | 5 | 6 | 7 | 8 | 9 | 10 |
| Je trouve cela difficile de reconnaître quand un patient est en détresse psychologique. | O | O | O | O | O | O | O | O | O | O | O |
| J'ai besoin de plus de formation à ce sujet. | O | O | O | O | O | O | O | O | O | O | O |
| Je n'ai pas de problèmes ou de difficultés qui m'empêchent de bien le faire (reconnaître et reconnaître les besoins en matière de détresse psychologique). | O | O | O | O | O | O | O | O | O | O | O |
| Je n'ai pas le temps. | O | O | O | O | O | O | O | O | O | O | O |
| Il n'y a pas d’endroit où référer les patients une fois qu'on a identifié leur détresse psychologique. | O | O | O | O | O | O | O | O | O | O | O |
| J'arrive à identifier et répondre à leurs besoins efficacement. | O | O | O | O | O | O | O | O | O | O | O |
| J'ai du mal à le faire parce que c'est trop éprouvant pour moi. | O | O | O | O | O | O | O | O | O | O | O |
| Je n'ai pas les compétences requises. | O | O | O | O | O | O | O | O | O | O | O |
| Je bénéficie d'un bon soutien de la part de mon unité de soins pour m'aider à y parvenir. | O | O | O | O | O | O | O | O | O | O | O |

8. En réfléchissant maintenant aux **facteurs qui vous aideraient** à identifier et à répondre aux besoins des personnes atteintes de maladie rénale chronique en détresse psychologique, veuillez indiquer dans quelle mesure chacun des éléments énumérés ci-dessous vous serait utile. *(Veuillez cocher, svp)*

|  | **Pas du tout utile** | | |  |  |  |  |  | **Très utile** | | |
| --- | --- | --- | --- | --- | --- | --- | --- | --- | --- | --- | --- |
|  | 0 | 1 | 2 | 3 | 4 | 5 | 6 | 7 | 8 | 9 | 10 |
| Des plages horaires plus longues avec les patients. | O | O | O | O | O | O | O | O | O | O | O |
| Un outil de dépistage pour les patients qui ont besoin de soutien psychologique et psychosocial. | O | O | O | O | O | O | O | O | O | O | O |
| Un.e infirmièr.e spécialisé.e (ex. en santé mentale) à qui on pourrait référer les patients. | O | O | O | O | O | O | O | O | O | O | O |
| De la formation sur la meilleure façon d'approcher et d’aider les patients à ce sujet. | O | O | O | O | O | O | O | O | O | O | O |
| Plus de reconnaissance à travers le salaire ou des opportunités de promotion ou autres responsabilités professionnelles. | O | O | O | O | O | O | O | O | O | O | O |
| Des interventions disponibles que je peux suggérer aux patients, par exemple le soutien par les pairs, des groupes de discussion, etc. | O | O | O | O | O | O | O | O | O | O | O |
| Accès à un.e psychologue spécialisé.e pour les patients. | O | O | O | O | O | O | O | O | O | O | O |
| La possibilité pour les patients de toujours voir la ou le même infirmièr.e dans chaque clinique ou RDV de suivi. | O | O | O | O | O | O | O | O | O | O | O |
| Savoir où orienter les patients pour obtenir de l'aide. | O | O | O | O | O | O | O | O | O | O | O |
| Avoir accès à du support émotionnel pour le personnel clinico-administratif qui apporte du soutien émotionnel aux patients. | O | O | O | O | O | O | O | O | O | O | O |
| Savoir quels patients cibler en particulier et à quel moment. | O | O | O | O | O | O | O | O | O | O | O |

Veuillez indiquer tout autre facteur, non mentionné ci-dessus, que vous jugeriez utile.

|  |
| --- |

9. Quelles sont les 3 ou 4 choses qui aideraient le plus les services de néphrologie à mieux identifier et répondre aux besoins des personnes atteintes de maladie rénale chronique en détresse psychologique ? *(Veuillez les écrire ci-dessous)*

10. Quel est votre âge ? ________________________________

11. Êtes-vous un homme, une femme ou autre (précisez) ? ________________________________

 12. Quelle est la date de naissance de votre mère ? (Veuillez indiquer jj/mm/aaaa).

*Cela nous aidera à regrouper les questionnaires anonymes que vous aurez rempli si jamais nous les faisons remplir à nouveau.*

________________________________

 13. Lequel des énoncés suivants décrit le mieux votre fonction actuelle ?

- Infirmier.e spécialisé.e
- Infirmier.e d’étage
- Infirmier.e de dialyse
- Diététicien.ne
- Psychologue
- Travailleur.euse social
- Coordonnateur.rice de soins
- Chef.fe de département
- Néphrologue
- Résident.e
- Agent.e administratif.ve
- Coordonnateur.rice d’unité ou connexe
- Gestionnaire
- Direction de la clientèle
- Direction des services multidisciplinaires
- Professionnel.le en génie biomédical ou connexe
- Autre: ________________________________________

 14. Depuis combien de temps travaillez-vous dans votre fonction actuelle au sein de cet hôpital ?

- Moins de 6 mois
- 6 à 12 mois
- 12+ mois à 2 ans
- 2+ ans à 3 ans
- 3+ ans à 5 ans
- 5+ ans à 10 ans
- 10+ ans à 20 ans
- 20+ ans à 30 ans
- Plus de 30 ans

 15. Quelle est la fréquence de vos contacts avec les personnes atteintes de maladie rénale chronique ?

- Tous les jours ouvrables
- La plupart des jours ouvrables
- 3-4 jours par semaine
- 1 fois par semaine
- Toutes les 2-3 semaines
- 1 fois par mois
- Moins d'une fois par mois
- Jamais

 16. Depuis combien de temps êtes-vous qualifié.e pour votre poste actuel ?

- Moins de 6 mois
- 7 à 12 mois
- 12+ mois à 2 ans
- 2+ ans à 3 ans
- 3+ ans à 5 ans
- 5+ ans à 10 ans
- 10+ ans à 20 ans
- 20+ ans à 30 ans
- 30 ans et plus

17. **Avez-vous reçu une formation** sur la manière d'identifier et de répondre aux besoins des personnes atteintes de maladie rénale chronique qui sont en détresse psychologique ? *(Cocher tous ceux qui s’appliquent).*

|  | Oui | Non |
| --- | --- | --- |
| Dans le cadre de ma formation professionnelle initiale. | O | O |
| Dans le cadre de la formation pour mon poste actuel. | O | O |
| Depuis que j’occupe mon poste actuel. | O | O |
| Au cours des 3 dernières années. | O | O |

 18. Enfin, seriez-vous prêt à nous aider à poursuivre nos recherches en participant à une discussion plus approfondie sur le sujet au cours des prochains mois ? Si oui, avez-vous une préférence entre les options suivantes *(cocher toutes celles qui vous conviendraient ou aucune préférence si applicable)*.

- Entrevue individuelle en visioconférence
- Entrevue individuelle en présentiel
- Entrevue de groupe en visioconférence
- Entrevue de groupe en présentiel
- Aucune préférence

Veuillez, s’il-vous-plait indiquer la meilleure façon de vous contacter :

Email/numéro de téléphone et à quel moment de la semaine vous contacter (si par téléphone).

**Autres commentaires**

Si vous avez **d’autres éléments importants à mentionner** ou discuter par rapport à la prise en charge des besoins émotionnels/de la détresse des personnes atteintes d’insuffisance rénale chronique, veuillez les mentionner ci-dessous.

**SUPPLEMENTAL FILE C**: Patient individual semi-structured interview guide

**Patient individual semi-structure interview guide**

**Accueil des participants**

- *Accueillir le participant et se présenter.*
- Merci pour le temps et l’intérêt dont vous faites preuve pour le projet, c’est vraiment apprécié.
- Comme je vous expliquais à notre dernière rencontre, **notre but** avec l’entrevue d’aujourd’hui c’est de comprendre **comment vous vivez avec votre maladie rénale**. On ne s’intéresse pas à ce que les autres personnes vivent avec leur maladie rénale, vraiment juste à votre réalité.
- S’il y a des questions auxquelles vous **n’êtes pas à l’aise de répondre**, vous n’avez pas à vous justifier, dites-le et on passera à la prochaine.
- Si vous ressentez le besoin de **prendre une pause** pendant l’entrevue, n’hésitez surtout pas à nous le dire et nous arrêterons le temps nécessaire.
- Pour qu’on puisse réécouter l’entrevue et en extraire l’information, l’**entrevue** d’aujourd’hui **va être enregistrée**, est-ce que ça vous convient toujours ?
- Toutes les informations qu'on recueille aujourd’hui resteront **confidentielle**, c’est-à-dire que votre nom ne sera jamais divulgué et qu’on ne pourra pas reconnaître vos commentaires ou partages. Cette discussion n’aura aucun impact sur vos soins médicaux personnels. Nous vous demandons votre opinion aujourd’hui pour améliorer le service.

**Questions d’entrevue**

**Légende** : *souligné* = questions essentielles. *Italique* = consignes pour interviewer

- D’abord, j’aimerais mieux vous connaître et que vous me parliez un peu de vous.
- C’est comment, pour vous, de vivre avec une maladie rénale ?
  - Comment la maladie rénale vous affecte dans la vie de tous les jours ?
  - Quels sont les symptômes physiques de votre maladie, s’il y en a ?
  - Comment la maladie affecte-t-elle votre moral ?
  - Comment est-ce que les gens autour de vous agissent par rapport à votre maladie rénale ?
- Pouvez-vous me décrire une journée typique dans votre vie ?
  - Je m’intéresse à des choses très concrètes, donc amenez-moi vraiment, pas à pas, à travers cette journée.
  - *Exemples de sous-questions si le participant élabore très peu*
    - Qui sont les personnes que vous rencontrez durant ces visites ?
    - Comment est-ce que vous vous sentez avant la dialyse ?
    - Comment se déroulent votre séance de dialyse ?
      - Que faites-vous durant la séance ?
      - Comment vous sentez-vous durant la séance ?
    - Comment vous sentez-vous après la dialyse ?
- Si vous avez déjà eu recours ou avez déjà fait des démarches pour avoir recours à des services de soutien en santé mentale, pourriez-vous me décrire votre expérience ?
- De quelle manière est-ce que le service de néphrologie pourrait vous aider à mieux vivre avec votre maladie rénale ?

**Conclusion**

- Nous serions sur le point de termine l’entrevue, s’il y a quelque chose que vous auriez souhaité ajouter, il nous reste quelques minutes.
- Merci de votre participation au projet de recherche et de votre ouverture durant cette séance d’entrevue. *Valider le ressenti du participant et féliciter pour son parcours (si pertinent)*.
- Les informations que vous nous avez partagées resteront totalement confidentielles, comme nous en avons déjà discuté. Et si vous l’avez indiqué sur le FIC, vous serez tenu.e au courant des résultats une fois que nous aurons complété l’analyse de l’ensemble des entrevues avec les autres participants.
- Comment avez-vous trouvé l’expérience ? Avez-vous des commentaires à nous donner par rapport au déroulement de l’entrevue ? *Peuvent les faire parvenir par email ou nous rappeler au besoin.*
- N’hésiter pas si vous avez des commentaires ou si vous avez des questions de nous les envoyer par courriel à [rein@crchudequebec.ulaval.ca](mailto:rein@crchudequebec.ulaval.ca) ou à nous appeler au poste #16 857.
- *Souhaiter la bonne journée et raccompagner le participant.*

Consignes pour les interviewers

- **Préparation à l’entrevue**
  - Préparer à l’avance la salle d’entrevue pour qu’elle soit le plus conviviale possible et limiter les éléments potentiellement distracteurs/interruptions.
  - Proposer des collations et eau/jus au participant avant et après la rencontre.
  - Avoir une boite de mouchoir proche
  - Démarrer les 2 enregistreurs.
- **Durant l’entrevue**
  - Laisser les participants s’exprimer avec le moins d’interventions possibles.
  - Les silences sont riches, donc il faut leurs laisser place.
  - Rester le plus neutre possible (ne pas réagir aux propos du participant) tout en étant à l’écoute et empathique.
  - Désigner le ou la participante comme : une personne avec une maladie rénale. Faire attention de ne pas avoir recours au terme *patient* ou *atteint* d’une maladie rénale pour les connotations qu’ont ces termes.
- **En cas de réaction émotionnel du participant**
  - Si le patient à la larme à l’œil, la gorge serrée, renifle ou pleurs, lui laisser le temps et l’espace de vivre son émotion. Il se pourrait bien que le participant continue tout de même à parler ou prenne un moment de silence.
  - On peut aussi nommer au participant qu’on voit que ça le touche et peut être demander ce qui le rend émotionnel. Peut mener à rendre la discussion encore plus riche.
  - Si vous voyez que les émotions vécus par le participant l’empêchent de répondre aux questions, vous pouvez lui proposer de prendre une pause, lui proposer des mouchoirs et de l’eau. Laisser le temps nécessaire au patient. Reprendre quand le patient est prêt.
  - Rappel : Le patient à le droit d’arrêter l’entrevue à n’importe quel moment et retirer son consentement. **
  - À la fin de l’entrevue, spécifiquement pour les participants très émotionnels, lui demander s'il veut recevoir de l’aide. Si oui, lui proposer de faire une demande au travailleuses sociales ou lui offrir notre liste de ressources.

**SUPPLEMENTAL FILE D:** Staff focus-group and individual interview guides

**Staff focus-group interview guide**

**Accueil des participants**

- Bonjour à tous !
- Merci à tous d’avoir accepté de nous rencontrer aujourd’hui, de vous être déplacés, etc. Je me nomme ____ (modérateur) et voici ________ (assistant aux notes). La rencontre d’aujourd’hui a pour but d’échanger sur vos expériences en tant que membre du personnel (clinique ou administratif selon le groupe) appelé à travailler avec ou pour la clientèle de néphrologie.
- Notre but est de comprendre quels sont les besoins psychologiques et psychosociaux des patients atteints de maladie rénale chronique et quelles sont les barrières à l’implantation de programmes de soutien adaptés à ces besoins dans nos milieux cliniques. Nous souhaitons que ces informations soient le plus concrètes possible pour qu’elles puissent servir au développement de services pour ces patients.
- Mon rôle aujourd'hui est de guider la discussion en vous posant des questions. Il s’agit d’une discussion, donc si jamais durant la rencontre vous avez des questions, vous ne comprenez pas un mot ou le sens d’une question, surtout n’hésitez pas à nous arrêter et demander une précision. Il n’y a pas de bonne ou mauvaise réponse, seulement de différents points de vue. Vous pouvez être en désaccord avec les autres, mais on veut rester dans le respect d’opinions des autres. Si certaines personnes ont moins tendance à s’exprimer spontanément, il se peut que je leur demande leur point de vue. En revanche, vous n’êtes jamais contraint de répondre à une question.
- La rencontre d’aujourd’hui va être enregistrée, il est donc important qu’une seule personne parle à la fois et de parler assez fort. Soyez assurés que votre anonymat sera préservé pour toutes personnes ne faisant pas partie de l’équipe de recherche.
- Dans ce même esprit, nous devons vous demander de respecter la confidentialité des autres participants. L’information partagée durant notre rencontre ne devra donc en aucun cas être divulguée en dehors du groupe.
- Finalement, on apprécierait si tous les téléphones pouvaient être fermés jusqu'à la fin de la discussion.
- J’aimerais commencer par un tour de table : Dites votre nom, votre pronom de choix si vous en avez un et un et quel poste vous occupez au sein du CHU de Québec.

**Éthique**

- L’ensemble des informations que vous nous partagerez aujourd’hui, de même que tous le fichiers audio et vidéo issus de notre entretien seront confidentiels.
- Nous allons nous intéresser uniquement à **votre** vécu et **votre** ressenti et non pas à celui d’autres personnes membres du personnel que vous côtoyez. Il n'y a donc pas de bonne ou mauvaise réponse. Je vais d’ailleurs vous encourager à donner les plus d’exemples concrets tirés de votre vie pour mieux comprendre ce que vous vivez.
- Si jamais vous ne comprenez pas la façon dont une question est formulée, n’hésitez pas à m’interrompre pour demander des clarifications.
- Finalement, si vous n’êtes pas à l’aise de répondre à une question, vous n’avez pas à vous justifier ou vous sentir mal. Simplement dire que vous préférez ne pas répondre et nous passerons à la prochaine.
- Et si jamais vous souhaitez terminer l’entretien au cours de rencontre, nous arrêterons l’enregistrement et pourrons discuter à savoir si vous acceptez ou non que l’on conserve ce qui a été enregistré.

**Croyances et connaissances**

- Comment est-ce que vous définiriez la détresse psychologique ?
- Croyez-vous que les patients suivis en néphrologie vivent des difficultés psychologiques ? Si oui, lesquels ?
- À quel moment dans la trajectoire de soins des patients, allant du diagnostic à la fin de vie, croyez-vous qu’il existe le plus de besoins en termes de soutien psychologique ?
- D’après vous, qu’est-ce qui pourrait être la cause de ces difficultés ?
- Selon vous, quelles sont les conséquences de la détresse psychologique vécue par les patient.es ?

**Prise en charge et ressources disponibles**

- Pourriez-vous me donner un exemple d’un moment où vous avez perçu de la détresse psychologique chez un.e patient.e ?
- Comment avez-vous réagi ou êtes intervenu ?
- Connaissez-vous des ressources disponibles pour soutenir les patients avec maladie rénale vivant de la détresse psychologique ?
  - En avez-vous déjà présentées à un.e patient.e ?
  - Comment est-ce que cela s’est déroulé ?
  - Aviez-vous eu un retour de la part du patient ayant utilisé ce service ?

**Barrières à l’implantation de nouvelles initiatives**

- Avez-vous déjà été témoins d’une initiative visant à améliorer le bien-être des patients de néphrologie ?
  - Si oui, quels étaient les bons coups de cette initiative ?
  - Comment croyez-vous que celle-ci aurait pu être bonifiée ?
- Selon vous, pourquoi l’implantation d’interventions ou de programme pour soutenir les patients vivant avec une maladie rénale chronique est limitée ?
- Avec tout ce qu’on a discuté aujourd’hui, comment pourrait-on améliorer le bien-être des patients en néphrologie ?

***Questions plus spécifiques pour le personnel clinique en néphrologie***

- Êtes-vous ou avez-vous déjà été témoin de détresse psychologique vécue par les patients suivis en néphrologie ?
- Comment est-ce que la détresse que vous percevez chez les patients atteints de maladie rénale chronique affecte votre moral ou votre satisfaction au travail ?
- De quelle manière est-ce que le service de néphrologie pourrait **vous** aider à adresser les émotions vécues en lien avec la détresse des patients ?

**Demandez à l’assistant s’il veut poser une question**

**Résumé de la discussion**

- Donc pour résumer notre discussion, … (par modérateur ou assistant)
- Est-ce que ce résumé vous semble adéquat ?
- Avons-nous passé à côté d’un sujet que vous voulez mentionner ?

**Conclusion du focus-group**

- Merci encore une fois pour votre participation ! Votre implication nous aidera grandement à cerner les réels besoins (psy) des patients atteints de maladie rénale et nous permettra de mettre en place un meilleur système de soutien (psy).
- Vous pouvez quitter si vous le désirez, mais n’hésiter pas si vous avez des commentaires ou si vous avez des questions de nous les envoyer par courriel à [rein@crchudequebec.ulaval.ca](mailto:rein@crchudequebec.ulaval.ca) ou à nous appeler au poste #16 857.

**Partie subsidiaire du groupe de discussion** :

1. Inviter les patients à *débriefer* de l’expérience qu’ils viennent de vivre.
2. Ventiler sur la charge émotionnelle que le *focus-group* leur a créée.
3. Advenant qu’un.e participant.e mentionne une grande détresse en lien avec la discussion, aviser l’un des médecins ou psychologue membre de l’équipe de recherche pour évaluer le participant. Référence à la ressource appropriée (ligne d’écoute, urgence, etc) selon le jugement du professionnel.

Merci de votre participation !

**Référence**

Twohig PL, Putnam W: Group interviews in primary care research: advancing the state of the art or ritualized research?. Fam Pract. 2002, 19: 278-284. 10.1093/fampra/19.3.278.

**Staff individual semi-structure interview guide**

**Accueil des participants**

- Bonjour Monsieur, Madame, Mx _________________________,
- Comment allez-vous ?
- (Si entrevue virtuelle : Vous n’avez pas eu trop de mal à vous connecter ? Parfait.)
- Je me présente, je m’appelle _____________________, je suis membre de l’équipe de recherche du Dr Agharazii. Je vais d’abord vous expliquer le déroulement de la séance, puis je démarrerai l’enregistrement de l’entretien.
- D’abord, je tiens à vous remercier de votre ouverture à participer à notre projet de recherche.
- **L’objectif,** comme nous l’avions discuté à la première visite, est de préciser quels sont les besoins psychologiques et psychosociaux des personnes atteintes de maladie rénale chronique, et quels sont les obstacles à l’implantation de services de soutien psychologique et psychosocial adapté à ces besoins.
- Pendant la visite d’aujourd’hui, je vais vous poser une série de questions, mais ce sera surtout l’opportunité pour vous de vous exprimer sur votre expérience face à la détresse psychologique vécue par les patients suivis en néphrologie.
- J’en profite pour vous présenter ma collègue __________________ qui assistera à la séance pour prendre des notes et nous aider dans l’animation de la discussion.
- **Éthique**
  - L’ensemble des informations que vous nous partagerez aujourd’hui, de même que les fichiers audios et vidéos issus de notre entretien seront confidentiels.
  - Nous allons nous intéresser uniquement à **votre** vécu et **votre** ressenti et non pas à celui d’autres personnes membres du personnel que vous côtoyez. Il n'y a donc pas de bonne ou mauvaise réponse. Je vais d’ailleurs vous encourager à donner les plus d’exemples concrets tirés de votre vie pour mieux comprendre ce que vous vivez.
  - Si jamais vous ne comprenez pas la façon dont une question est formulée, n’hésitez pas à m’interrompre pour demander des clarifications.
  - Finalement, si vous n’êtes pas à l’aise de répondre à une question, vous n’avez pas à vous justifier ou vous sentir mal. Simplement dire que vous préférez ne pas répondre et nous passerons à la prochaine.
  - Et si jamais vous souhaitez terminer l’entretien au cours de rencontre, nous arrêterons l’enregistrement et pourrons discuter à savoir si vous acceptez ou non que l’on conserve ce qui a été enregistré.

Nous allons commencer l’entrevue, si vous êtes prêt.e :

- **Croyances et connaissances**
  - Comment est-ce que vous définiriez la détresse psychologique ?
  - Croyez-vous que les patients suivis en néphrologie vivent des difficultés psychologiques ? Si oui, lesquels ?
  - À quel moment dans la trajectoire de soins des patients, allant du diagnostic à la fin de vie, croyez-vous qu’il existe le plus de besoins en termes de soutien psychologique ?
  - D’après vous, qu’est-ce qui pourrait être la cause de ces difficultés ?
  - Selon vous, quelles sont les conséquences de la détresse psychologique vécue par les patient.es ?
- **Prise en charge et ressources disponibles**
- Pourriez-vous me donner un exemple d’un moment où vous avez perçu de la détresse psychologique chez un.e patient.e ?
- Comment avez-vous réagi ou êtes intervenu ?
- Connaissez-vous des ressources disponibles pour soutenir les patients avec maladie rénale vivant de la détresse psychologique ?
  - En avez-vous déjà présentées à un patient ?
  - Comment est-ce que cela s’est déroulé ?
  - Aviez-vous eu un retour de la part du patient ayant utilisé ce service ?
- **Barrières à l’implantation de nouvelles initiatives**
  - Avez-vous déjà été témoins d’une initiative visant à améliorer le bien-être des patients de néphrologie ?
  - Si oui, quels étaient les bons coups de cette initiative ?
  - Comment croyez-vous que celle-ci aurait pu être bonifiée ?
  - Selon vous, pourquoi l’implantation d’interventions ou de programme pour soutenir les patients vivant avec une maladie rénale chronique est limitée ?
  - Avec tout ce qu’on a discuté aujourd’hui, comment pourrait-on améliorer le bien-être moral des patients en néphrologie ?
- ***Questions plus spécifiques pour le personnel clinique en néphrologie***
- Êtes-vous ou avez-vous déjà été témoin de détresse psychologique vécue par les patients suivis en néphrologie ?
- Comment est-ce que la détresse que vous percevez chez les patients atteints de maladie rénale chronique affecte votre moral ou votre satisfaction au travail ?
- De quelle manière est-ce que le service de néphrologie pourrait **vous** aider à adresser les émotions vécues en lien avec la détresse des patients ?

Nous serions sur le point de termine l’entrevue, avez-vous d’autres commentaires à ajouter en lien avec les sujets abordés précédemment ?

- Merci énormément de votre participation au projet de recherche et de votre ouverture durant cette séance d’entrevue.
- Comment avez-vous trouvé l’expérience ?
- S’il y a quelque chose que vous auriez vraiment souhaité mentionner, il nous reste quelques minutes. Aviez-vous des questions par rapport à la suite des choses ?
- Vous pouvez quitter si vous le désirez, mais n’hésiter pas si vous avez des commentaires ou si vous avez des questions de nous les envoyer par courriel à [rein@crchudequebec.ulaval.ca](mailto:rein@crchudequebec.ulaval.ca) ou à nous appeler au poste #16 857.
- Sinon, je vous souhaite une bonne fin de journée.
